# Insulin-Like Growth Factor Binding Proteins in Autoimmune Diseases

**DOI:** 10.3389/fendo.2018.00499

**Published:** 2018-08-30

**Authors:** Huihua Ding, Tianfu Wu

**Affiliations:** ^1^Department of Rheumatology, Renji Hospital, Shanghai Jiao Tong University School of Medicine, Shanghai, China; ^2^Department of Biomedical Engineering, University of Houston, Houston, TX, United States

**Keywords:** IGFBPs, autoimmune diseases, biomarkers, therapeutic targets, metabolism

## Abstract

Insulin-like growth factor binding proteins (IGFBPs) are a family of proteins binding to Insulin-like growth factors (IGFs), generally including IGFBP1, IGFBP2, IGFBP3, IGFBP4, IGFBP5, and IGFBP6. The biological functions of IGFBPs can be classified as IGFs-dependent actions and IGFs-independent effects. In this review, we will discuss the structure and function of various IGFBPs, particularly IGFBPs as potential emerging biomarkers and therapeutic targets in various autoimmune diseases, and the possible mechanisms by which IGFBPs act on the pathogenesis of autoimmune diseases.

## Introduction

Insulin-like growth factor binding proteins (IGFBPs) are a group of secreted proteins which serve as transport proteins for insulin-like growth factors (IGFs) with high affinity, regulating the bioavailability and function of IGFs. The IGFBP family consists of six IGFBPs, namely IGFBP1 through IGFBP6, however other proteins with low binding affinity to IGFs were incorrectly named as IGFBP7, IGFBP8, IGFBP9 etc., a consequence of belonging to the IGFBP-related protein (IGFBP-rPs) family (1, 2). Due to the conserved protein structure and high binding affinity to IGFs, only IGFBP1 through IGFBP6 are considered true IGFBPs. The eponymous function of IGFBPs is achieved through binding to IGFs thus regulating their biological activity; however, in pathological conditions, especially under cancer status, the role of IGFBPs in IGF-independent pathways has prompted increasing attention (3). In autoimmune diseases, IGFBPs are also known to play a role in the development of diseases both through IGF-dependent and IGF-independent pathologies (4, 5). In this review, we focus on the six true IGFBPs and their roles in autoimmune diseases, including their potential roles as biomarkers and therapeutic targets.

## The IGFBP family: structure and function

**Figure 1:**
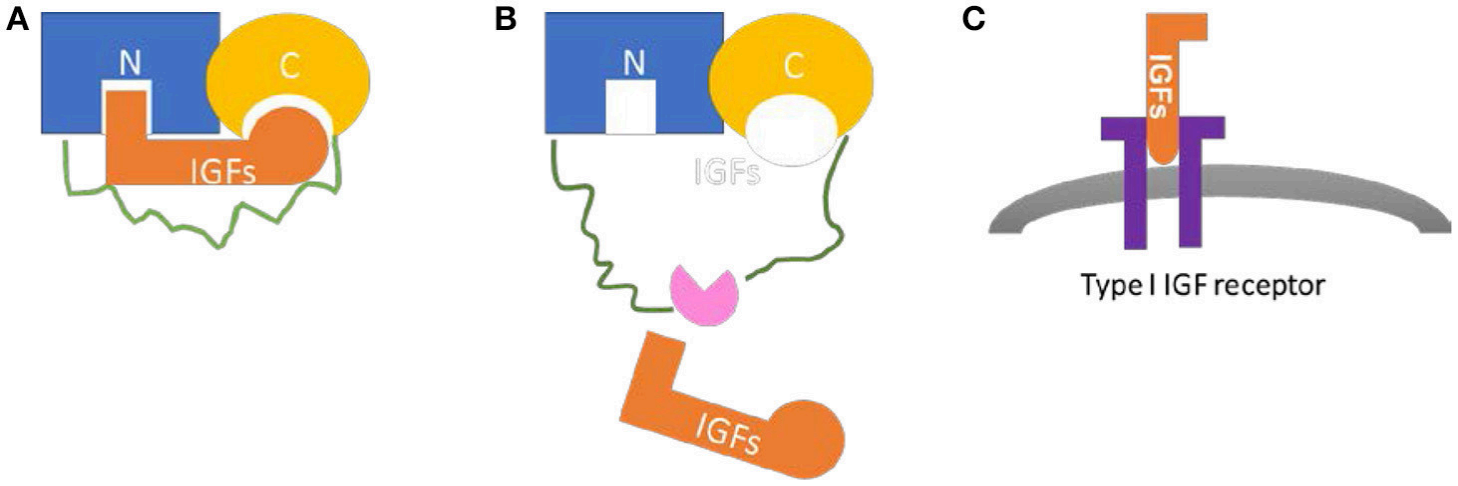
Common structure and biological function of IGFBPs. **(A)** The N-terminal region (blue) and the C-terminal region (yellow) are connected by a linker region (green). Both N-domain and C-domain contains a binding site for IGFs (orange). **(B)** The proteolysis of IGFBPs by various proteases (pink) occurs in the linker region or other post-translational modifications, may result in IGFs release. **(C)** Once released from IGFBPs, IGFs bind to IGF receptors (purple) to exert their physiological effects.

The IGFBP family comprises six structurally similar proteins with high affinity to IGFs. The six proteins, with a mass of ~24 to 50 kDa (216–289 amino acids), share a highly conserved structure with three domains of similar sizes: the conserved N-terminal cysteine rich region and the C-terminal cysteine rich region connected by a less structural and less conserved linker region (6, 7) (Figure 1). High-affinity IGFs binding capacity requires both the N- and C-terminals, with relative IGFs-binding affinities differ among IGFBPs (6). The linker domain is highly variable and unique to each IGFBPs; It's involved in the proteolysis of IGFBPs by several proteases, with cleavage sites located in the domain (7–9). The proteolysis of IGFBPs results in IGFs release, which provides a mechanism for the post-translational modification of IGFBPs. Other post-transcriptional modifications, such as glycosylation and phosphorylation, have also been implicated in this region which can modulate the function of IGFBPs (10–12). The linker domain also indirectly plays a role in high affinity IGF binding through inter-domain movement control during ligand binding (13).

The biological function of IGFBPs can be classified as IGFs-dependent actions and IGFs-independent effects. Both IGF-I and IGF-II are protein hormones structurally and functionally similar to insulin, which play extensive roles in growth and development. When binding to insulin-like growth factor 1 receptor (IGF1R), IGFs activate the intracellular IGF signaling pathway and promote cell proliferation and differentiation as well as inhibit cell apoptosis (14, 15). In blood, most IGFs (~75%) circulate as a 150 kDa ternary complex containing IGFs bound to IGFBP3 and a glycoprotein called acid labile subunit (ALS) (16–18). The rest of IGFs (~25%) are bound to the six IGFBPs to form a 50 kDa binary complex. It is through this binding that IGFBPs regulate the bioavailability of IGF-I and IGF-II in a range of ways. Firstly, they transport and store IGFs in circulation such that IGFs are more stable with an extended half-life of several hours in ternary complex and several minutes in a binary complex (16). Secondly, IGFBPs modulate the action of IGFs in both positive and negative manners. Since IGFBPs have higher affinity to IGFs than IGF1R, the binding of IGFBPs to IGFs sequestrate IGFs from their receptors inhibiting the effects of IGFs (19–21). This inhibitory effect has been widely proved in the case of IGFBP4 in both *in vitro* and *in vivo* studies (22–24). On the other hand, some IGFBPs (IGFBP1, IGFBP3, IGFBP5) may stimulate IGF actions in certain circumstances.

The IGF independent action of IGFBPs include effects on cell adhesion and migration, cell growth and apoptosis (25, 19). IGFBP1 was reported to increase cell migration of Chinese hamster ovary (CHO) cells, which was mediated through the binding of RGD motif in C-terminal of IGFBP1 to α_5_β_1_ integrin (26). In human carotid plaques, the expression of IGFBP1 is significantly increased and IGFBP1 has been proven to stimulate smooth muscle cells proliferation through ERK1/2 activation (27). Both the intact and proteolyzed form of IGFBP3 have been demonstrated to have IGF-independent growth-stimulatory and inhibitory effects in several cell lines including a range of cancer cell lines (28–34). IGFBP3 also protects against retinal endothelial cell apoptosis through inhibition of TNF-α production (35). In the respiratory system, IGFBP3 treatment reduces airway inflammation and hyper-responsiveness via activation of IGFBP3 receptor pathway (36). IGFBP5 fragments were first shown to stimulate osteoblast mitogenesis in the absence of IGF-I and recombinant human IGFBP5 stimulated osteoblast proliferation without the aid of IGF-I (37–39). Using genetic engineering, Pell JM et al proved that IGFBP5 played an important role in cell proliferation and apoptosis both *in vitro* and *in vivo* via an IGF-independent mechanism (40, 41). Due to the different structures of various IGFBPs, the expression, binding kinetics and dynamics may vary in a dose-dependent manner at temporal and spatial levels and may all contribute to the specific functions of IGFBPs at different physiological situations.

## IGFBPs as biomarkers in autoimmune diseases

The past few decades have witnessed a growing interest in the exploration of biomarkers in human diseases. This growth is fueled by the biological functions of biomarkers as they can be used as diagnostic tool to improve the accuracy of diagnosis, possible early detection of diseases, monitoring markers allowing for elucidation of disease activity and complications. Furthermore, they can also be used as prognostic markers allowing for prediction of possible patient outcomes.

IGFBP family members have been indicated to be involved in the development and progression of tumors and may be useful prognostic biomarkers in various malignancies (3). Recent studies also validated IGFBPs' role in the diagnosis and prognosis prediction in some solid tumor including nasopharyngeal carcinoma, ovarian cancer, pancreatic cancer, etc. (42–46). Despite the huge development of IGFBPs as biomarkers in cancer, there have been a number of studies focusing on the utility of IGFBPs as biomarkers in autoimmune diseases (Table 1). Despite the advances of researches on IGFBPs as biomarkers in cancer, there have been several studies focusing on the utility of IGFBPs as biomarkers in autoimmune diseases (Table 1), most of which were investigating the diagnostic role of IGFBPs in the disease. While the potential role of IGFBPs as diagnostic biomarkers has been summarized below, the mechanism of action for these molecules has not been widely investigated. Here we summarized studies investigating the potential roles of IGFBPs in the diagnosis and monitor of autoimmune diseases.

**Table 1:** Summary of IGFBPs as biomarkers in autoimmune diseases.

**Autoimmune disease**	**GFBP1**	**IGFBP2**	**IGFBP3**	**IGFBP4**	**IGFBP5**	**IGFBP6**	**References**
Type I diabetes	↑	↑/↓	↑/–	↑		↓	(47–63)
Multiple sclerosis	↑/–	↑/–	↑/↓/–				(64–70)
Rheumatoid arthritis		↑	Synovial fluid ↑, Serum ↑/↓				(71–83)
Juvenile idiopathic arthritis			Synovial fluid ↓, Serum↓				(84–86)
Systemic lupus erythematosus		↑		↑			(87–90)
Systemic sclerosis			↑		↑		(91–93)
Inflammatory bowel disease		↑					(94)

*↑, means increased in serum; –, means no change; ↓, means decreased in serum*.

### Type 1 diabetes mellitus (T1DM)

Type 1 diabetes mellitus (T1DM) is an organ-specific autoimmune disease in which the insulin-producing pancreatic β cells are destroyed by the immune system. Due to the close relationship to insulin, the system of IGF and its binding proteins has been first explored as biomarkers in diabetes, especially T1DM. Serum IGFBP1 levels were consistently reported to be increased in T1DM population including prepubertal and pubertal individuals (47–52). In children with new onset diabetes, serum IGFBP1 served as a differential diagnostic marker for T1DM and T2DM (50). In young T1DM patients, IGFBP1 levels were increased independent of pubertal status (52). Besides being a diagnostic marker, serum IGFBP1 level was a good indicator of complications related to T1DM. In T1DM patients complicated with microalbuminuria, serum IGFBP1 level has been reported to be significantly increased (53). It's also a biomarker for diabetic retinopathy in T1DM but not T2DM patients (54). Serum IGFBP2 levels were also reported as an increased biomarker in T1DM (53, 55). In a recent study, Zhi et al. used a proteomic approach to identify IGFBP2 as a potential diagnostic biomarker for T1DM (56). However, in diabetic retinopathy, IGFBP2 levels have been reported to be either increased or decreased in T1DM patients (51, 54). IGFBP3, the most abundant type of IGFBPs, has been widely studied as biomarker in T1DM. In T1DM patients, serum IGFBP3 levels have reported to be significantly decreased in different populations, which was partially explained by increased proteolysis (47, 48, 53, 55, 56). However, in T1DM women during pregnancy, serum IGFBP3 levels have been implicated to either increase or decrease, which might be explained by the different testing time period (57, 58). IGFBP3 level also worked as a severity marker for T1DM. It correlated with HbA1c, total cholesterol, and LDL-cholesterol levels (59) and inversely correlated with blood pressure (60). Besides, patients complicated with autoimmune thyroiditis and coeliac disease had significantly lower serum concentration of IGFBP3 (61). More interestingly, IGFBP3 levels were significantly increased in the tears of diabetic patients, indicating the potential contribution to pathogenesis of ocular complications in diabetes (62). IGFBP4 was only reported in one study to be significantly increased in prepubertal T1DM children (47). More recently, higher IGFBP4 fragment levels was reported to be associated with cardiovascular mortality rates in T1DM patients, which made it a potential prognostic biomarker (63). Decreased IGFBP6 was reported to be related with diabetic retinopathy in both T1DM and T2DM (54).

### Multiple sclerosis (MS)

Multiple sclerosis (MS) is a demyelinating autoimmune disease in which the immune system damages the myelin sheath of the nerve cells in the central nervous system. The first study investigating IGFBPs concentrations in MS patients revealed no differences in concentrations of IGFBP1, IGFBP2, and IGFBP3 in circulation and cerebrospinal fluid between MS patients and controls (64). Later on, with the enlargement of study population size, Al-Temaimi et al. reported an increased IGFBP1 level in female MS patients (65). Consistent with this, IGFBP1 as well as IGFBP6 were proven to be overexpressed in oligodendrocytes at the edges of demyelinated plaques, indicating a pathogenic role of them in the development of MS (66). Other studies focused on the serum concentration of IGFBP3 in MS, but the results lacked consistency (67–70). This inconsistency of IGFBP3 levels in MS patients may be due to the differences of sample size and patients' demographic characteristics. However, IGFBP3 level correlated with disease severity score, relapsing-remitting disease pattern and treatment strategy, indicating the involvement of IGFBP3 in the pathogenesis of MS (67, 69, 70). Serum IGFBP2 was reported to be increased in MS patients compared to healthy control (69).

### Rheumatoid arthritis (RA)

Rheumatoid arthritis (RA) is systemic inflammatory disease that primarily affects the joints. The profile of IGFBPs were first characterized in the synovial fluid of RA patients, in which IGFBP2, IGFBP3, and IGFBP4 levels were significantly elevated compared to normal individuals or osteoarthritis patients (71–74). IGFBP3 levels in the synovial fluid of RA patients correlated with systemic C-reactive protein (CRP) levels, indicating the involvement of IGFBP3 in inflammation (73). Unlike the synovial fluid, IGFBPs profiling in circulation of RA patients remains controversial. Early studies demonstrated a decreased serum level of IGFBP3 in RA patients (73, 75, 76), which correlated with habitual exercise level (75). However, other groups didn't observe the effect of dynamic exercise on serum IGFBP3 (77, 78). Toussirot et al. compared serum IGFBP3 levels in corticosteroid-treated RA patients, non-RA patients under corticosteroids treatment, and healthy population (79). No significant differences were observed. More recently, a number of studies have demonstrated an increased IGFBP3 levels in RA patients, which correlated with serum CRP level (80–82). The inconsistency of circulating IGFBP3 levels in RA patients could be due to the heterogeneity of RA population as well as differences in disease status and treatment since the treatment strategy for RA has been changed dramatically over time. Other than IGFBP3, serum IGFBP2 and IGFBP1 have been reported to increase in RA population (81, 83). In juvenile idiopathic arthritis, the expression of IGFBP3 was decreased both in the circulation and synovial fluid (84–86).

### Systemic autoimmune diseases

Systemic autoimmune diseases such as systemic lupus erythematosus (SLE), systemic sclerosis, inflammatory bowel disease and idiopathic pulmonary fibrosis have also been reported to be associated with IGFBPs (87–91, 94, 95). In SLE, IGFBP2, IGFBP4 and IGFBP6 were discovered by array-based proteomic screening as diagnostic biomarkers for lupus (89). Later validation studies using a larger study population confirmed the role of IGFBP2 as a diagnostic biomarker for SLE as well as lupus nephritis (88, 90).

Moreover, IGFBP2 is a potential disease monitoring biomarker for renal function and renal histopathologic changes in lupus nephritis (88). Another SLE marker, IGFBP4, was mainly increased in lupus nephritis patients, which makes it a good indicator for renal pathology chronicity changes (87). In systemic sclerosis, serum IGF-1 and IGFBP-3 levels were significantly elevated compared to SLE patients or healthy control (91). Yasuoka et al. demonstrated the overexpression of IGFBP5 in animal models of systemic sclerosis could induce fibrosis in the lung and the skin (92, 93). In inflammatory bowel disease patients, serum proteome profiling revealed elevated IGFBP2 levels in untreated patients and suppressed by steroids treatment, indicating the pro-inflammatory effect of IGFBP2 (94). Serum IGFBP1 and IGFBP2 are elevated in idiopathic pulmonary fibrosis patients and IGFBP2 level was significantly reduced by anti-fibrotic therapy (95).

All these studies suggest that IGFBPs are indicative of disease activity, although larger cohort of patients are still needed to validate these findings. Nevertheless, it is expected that these IGFBPs molecules may be involved in the pathogenesis of these autoimmune diseases and may serve as disease biomarkers to monitor flares or track drug responses.

## IGFBPs in autoimmune diseases: possible mechanisms

Although IGFBPs have been proved as potential biomarkers in a variety of autoimmune diseases, the underlying mechanism remains unveiled. Given that the biological function of IGFBPs can be divided into IGFs-dependent and independent effects, the underlying mechanism of IGFBPs in autoimmune diseases can also be divided into IGFs-dependent and IGFs-independent mechanisms. Immune regulation of IGF-I has been reviewed in detail elsewhere (96). A recent genome-wide association study (GWAS) in SLE patients suggested that serum IGF-1 levels were increased with SLE disease severity, and SLE may be affected by a modulation of the IGF-1 signaling pathway and +3179G/A IGF-1R polymorphism (97).

The following studies suggested that IGFBPs have direct effects on immunity and inflammation. Peripheral blood mononuclear cells (PBMC) mainly consist of lymphocytes and monocytes, representing cells of both the innate and adaptive immune systems. Early study demonstrated an inhibitory effect of IGFBP1 on the proliferation of activated PBMCs (98). However, an increase of IGFBP2 expression was indicated in activated human PBMCs and exogenous IGFBP2 treatment of enhanced the proliferation of human PBMCs, suggesting the involvement of IGFBP2 in lymphocyte proliferation (98). Intra-tracheal administration of IGFBP5 in mice induced mononuclear cell infiltration in the lung and *in vitro* IGFBP5 can stimulate migration of PBMCs (93). This monocyte/macrophage system contributes to autoimmune diseases and inflammation by phagocytosis and antigen presentation. Furthermore, several studies have demonstrated the impact of different IGFBPs on monocyte/macrophage. An increase of IGFBP3 expression was first reported to be related with increased monocyte apoptosis stimulated by lipopolysaccharide, which was proved to be IGF independent (99). Recently, IGFBP3 was demonstrated to inhibit monocyte-endothelial cell adhesion through down-regulate ICAM-1 expression under hyperglycemic condition, which can inhibit retinal inflammation (100). In the RA synovium, IGFBP3 was produced by macrophage (82). In a mouse model of lung fibrosis, IGFBP5 induced migration of activated CD4+T cells and monocytes, indicating a chemoattractant activity of IGFBP5 for immune cells (101). In the same model, monocytes treated with IGFBP5 acquired a mesenchymal phenotype *in vitro* and *in vivo* (101). *in vitro* culture of human hematopoietic stem cells revealed an inhibitory effect of IGFBP3 and a stimulatory effect of IGFBP6 on the development of pro-B-cell, which might be IGF-1 dependent (102). IGFBP3 was also proved to support the development and maintenance of naïve CD8+ T cells, indicating the beneficial impact of IGFBP3 in maintaining a health immune system (103). IGFBP2 supplement can maintain vigorous hematopoietic cell expansion and CD34+ phenotype (104).

**Figure 2:**
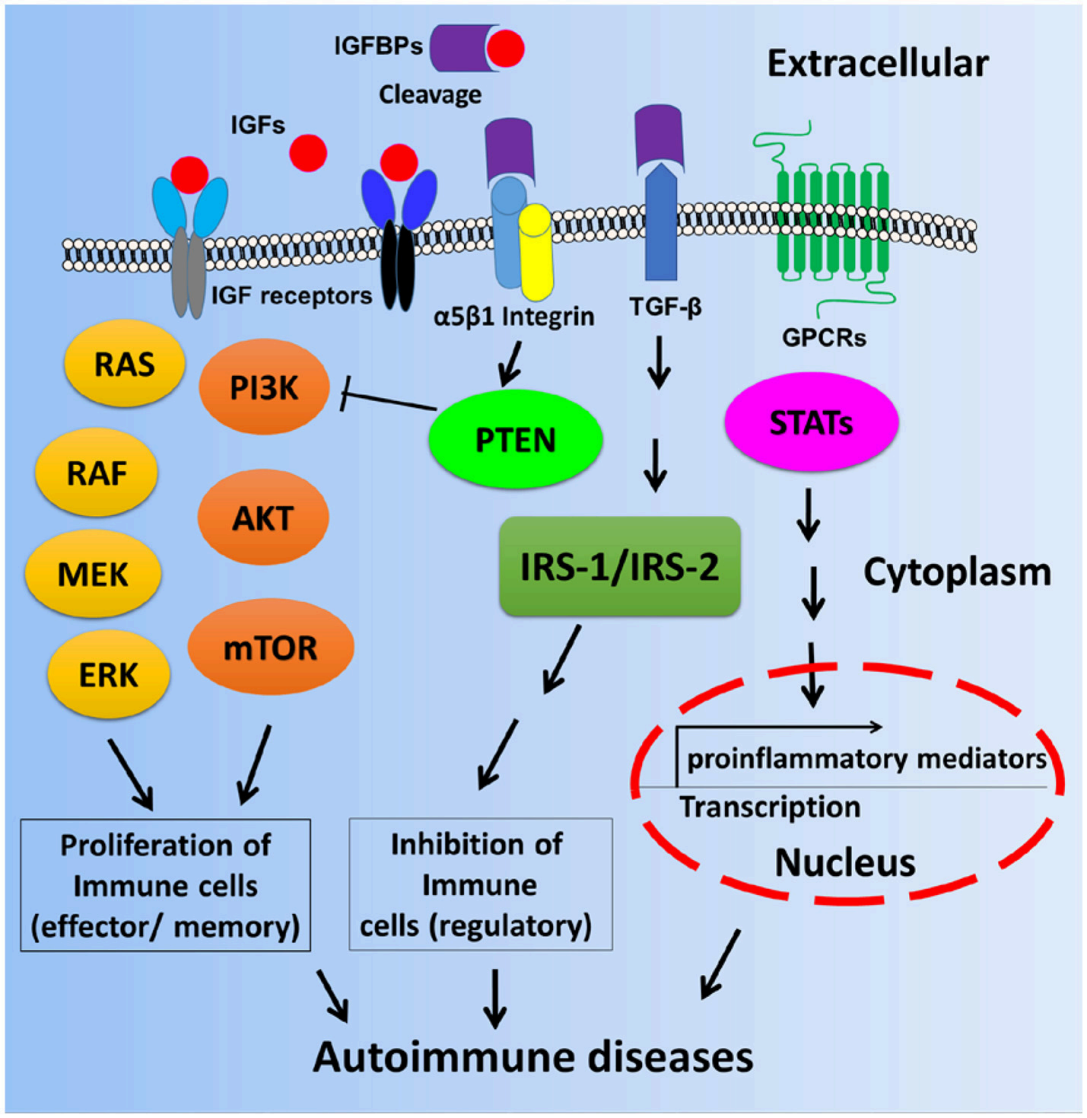
Scheme of possible mechanisms by which autoimmune diseases are mediated by IGF-IGFBP signaling pathways. Immune cell proliferation may be partly dictated by IGF-IGFBP pathway. The inhibition of regulatory immune cells or the control of gene transcription of proinflammatory cytokines may be mediated by IGF-independent pathways such as IGFBP3, TGF-β, and GPCR etc. STAT: Signal transducer and activator of transcription including STAT1, STAT2, STAT3, and STAT4.

These interesting findings suggest that IGFBPs may be involved in the pathogenesis of various autoimmune diseases, either via IGF-1 dependent signaling pathways or IGF-1 independent signaling pathways as depicted in Figure 2. Based on current knowledge, it is more apparent that IGF-IGFBP signaling axes may dictate cell proliferation and affect immune cell function or tissue damage. However, it is uncertain whether IGFBPs expression could impact proinflammatory pathways such cytokine production in the context of autoimmune diseases. Therefore, more delicate mechanistic studies are warranted in order to uncover the molecular and cellular basis and function of IGFBPs in autoimmune diseases.

## Look into the future

In the past decade, there has been a marked change in the way biomarker is discovered since the advent of high throughput techniques such as genomics, proteomics and metabolomics etc. The discovery of biomarkers used to be mechanism-driven, in which researchers choose candidate biomarkers based on their involvement in the pathogenesis of the disease to validate their role as biomarkers for disease diagnosis, disease monitoring, and prognostic evaluation. The investigation of IGFBPs in T1DM was a good example of this mechanism-driven strategy since IGFBP family is involved in insulin regulation, leading to the hypothesis that they might play a pathogenesis role in the development of T1DM. Most of the studies investigating the use of IGFBPs as biomarkers in autoimmune diseases in this review were based on the mechanism-driven strategy. Only two studies used proteomic technique to discover potential biomarkers for T1DM (56) and SLE (89). The untargeted global proteomic biomarker discovery is also called data-driven strategy, in which researchers use high throughput platforms to find potential biomarker from thousands of molecules regardless of their pathogenic role in the development of the disease (105).

Currently studies on IGFBPs role as biomarkers in autoimmune diseases have the following pitfalls. Firstly, most of the studies used conventional strategies instead of “omics” based high-throughput techniques. Future studies should adopt the more efficient and less biased strategy to discover biomarkers in autoimmune diseases. Secondly, almost all the studies adopted retrospective case-control study design, in which selection bias cannot be avoided. In the future, prospective studies are highly recommended to validate the existing IGFBPs role as biomarkers in autoimmune diseases. In addition, some IGFBPs such as IGFBP2 were proved to be increased in several different autoimmune diseases including T1DM, MS, RA, SLE, and IBD. It is not clear whether IGFBP2 is a general marker for autoimmunity or it is specific to certain kind of autoimmune diseases. A direct comparison of IGFBP2 levels in patients with these diseases should be helpful in clarifying this issue. Thirdly, the pre-analytical conditions including sample collection and processing for various studies were different, which partially explain the inconsistency of some studies on the validation of the same biomarker in different study population. To overcome this, a standardized protocol for sample handling or biobanking should benefit future studies.

## Author contributions

All authors listed have made a substantial, direct and intellectual contribution to the work, and approved it for publication.

### Conflict of interest statement

The authors declare that the research was conducted in the absence of any commercial or financial relationships that could be construed as a potential conflict of interest.
